# Supplementary material for: Interaction between Human Papillomavirus-Encoded E6 Protein and AurB Induces Cell Immortalization and Proliferation—A Potential Target of Intervention
Source: Cancers (Basel). 2023 Apr 25;15(9):2465. doi: 10.3390/cancers15092465 (PMC10177266; doi:10.3390/cancers15092465)
Supplement: Supplementary file 1 [file cancers-15-02465-s001.zip › cancers-2258899-supplementary.pdf]

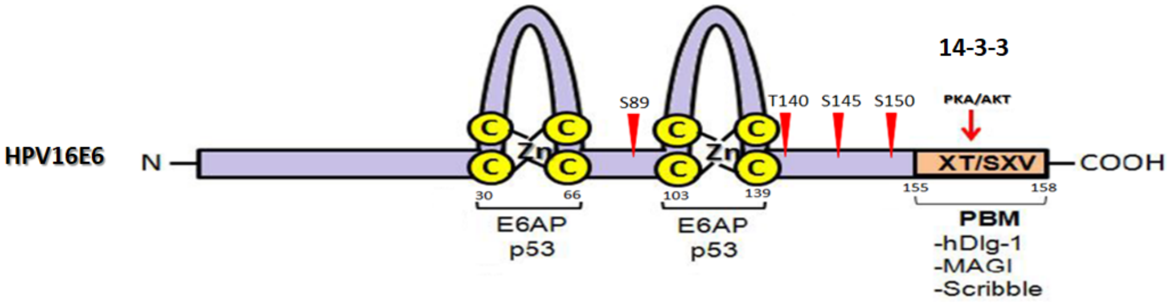

Phosphorylation Consensus motif

Aurora B $[R/K]_{1\text{-}3}$-X-[S/T]-ϕ

HPV E6	Site	Sequence
16	S89	YRHYCYSLYGTTLE
	S145	TGRCMSCCRSSRT
	S150	MSCCRSSRTRRETQ
	T140	NIRGRWTGRCMSCC
18	S59	LFVVYRDSIPHAACH

Figure S1. Schematic diagram showing the basic structure of cancer-causing HPV16E6 and potential amino acids recognised by Aurora B. E6 contains two major functional domains: two zinc (Zn) finger binding domains, the regions where E6 forms complex with E6AP and p53; and the unique dual-functional PDZ binding motif (PBM) at its extreme C-terminal end, the region where E6 binds to PDZ proteins (Dlg-1, MAGI and Scribble), and which is also a target for PKA/AKT phosphorylation, which switches binding affinity to the 14-3-3 protein family. The lower panel shows the Aurora B recognition motif $[R/K]_{1\text{-}3}$-X-[S/T] and the key amino acids within HPV16 and 18E6 predicted to be recognised by Aurora B: HPV16E6 at amino acids S89, T140, S145 and S150 of HPV16E6; HPV18E6 at amino acid S59.

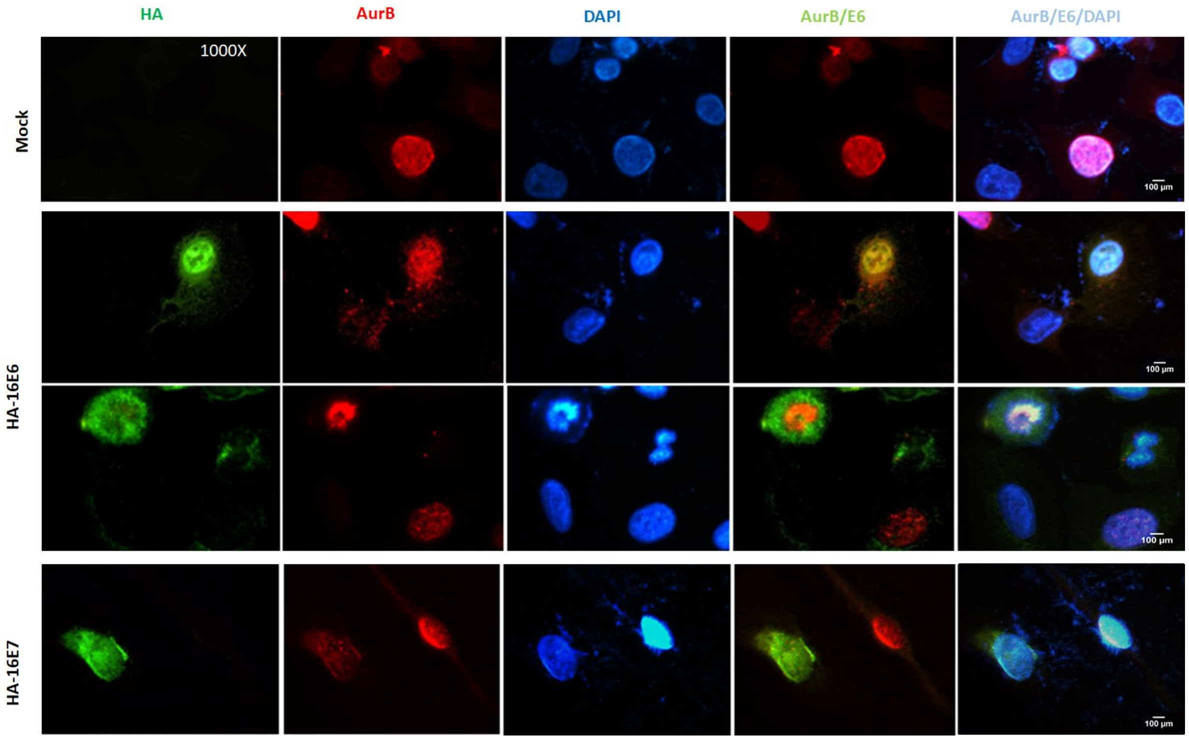

Figure S2. Subcellular colocalization of E6 with AurB predominantly in the nucleus. Immunofluorescence staining was performed to determine subcellular co-localisation of Aurora kinase B (AurB) and 16E6. HPV-null human osteosarcoma cells, U-2 OS were either mock-transfected or transfected with HA-tagged HPV-16E6 (HA-16E6) or E7 (HA-16E7). After 24 hrs, the cells were stained with HA (green) and AurB-specific (red) antibodies. Note the nuclear co-localisation of HPV16E6 in asynchronous U-2 OS cells, and co-localisation of AurB with E6 in mitotic cells (pointed by red arrows).

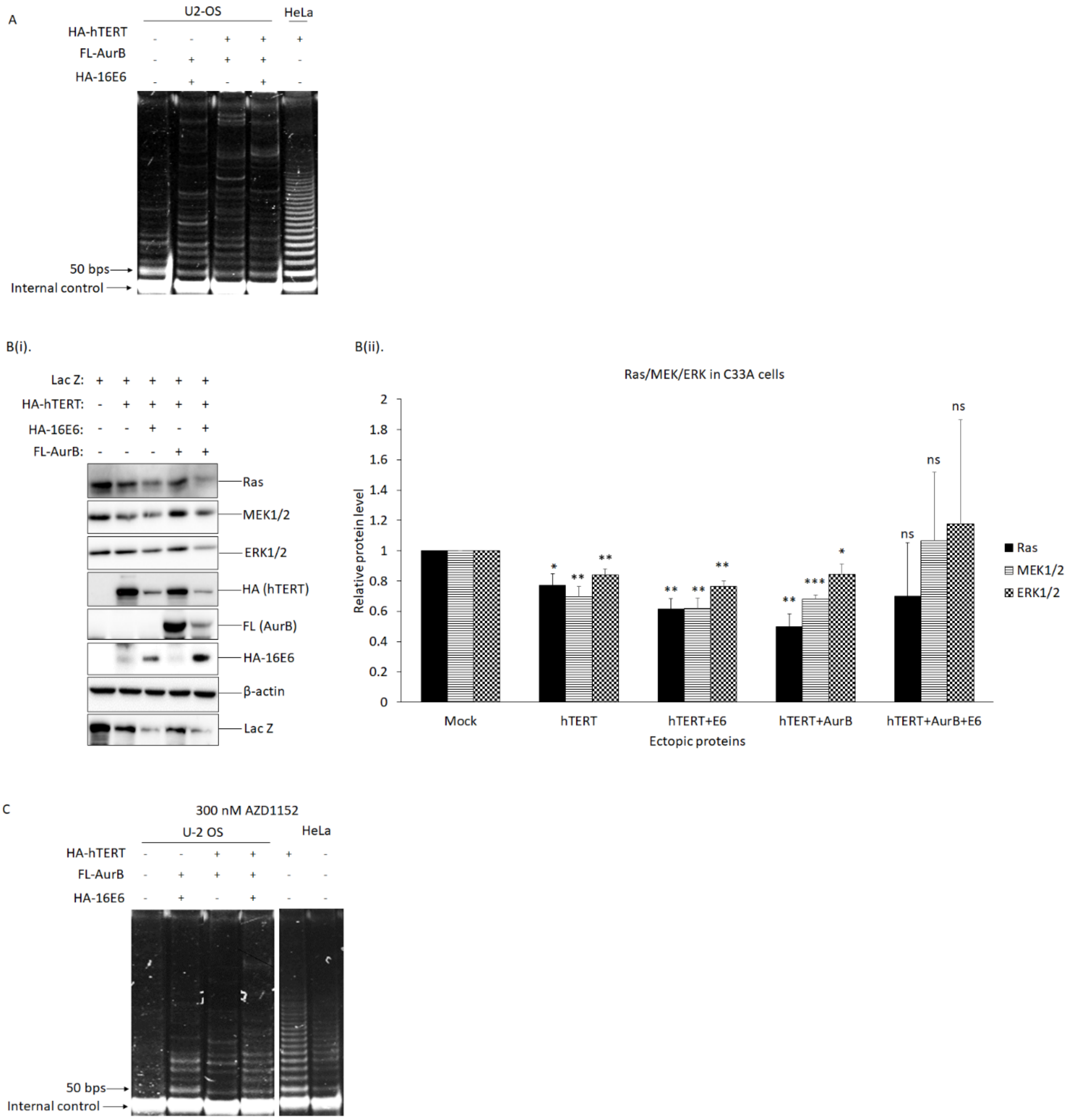

Figure S3. AurB-E6 did not disrupt telomerase activity, but perturbed Ras/MEK/ERK axis. A. The level of telomerase activity was measured using a PCR-based TRAPaze telomerase detection assay. Cell lysate of U2-OS cells transfected with HA-tagged hTERT alone, or with HA-tagged 16E and/or Flag-tagged AurB were extracted. Cell extract of HeLa cells was included as a positive control. **B(i).** The levels of members of the Ras/MEK/ERK axis were examined upon co-overexpression of Aurora B (AurB) and HPV16E6 in C33A cells. C33A cells were transiently transfected with either HA-tagged hTERT (HA-hTERT) alone, or co-transfected with HA-tagged HPV16E6 (HA-16E6), HA-tagged or Flag-tagged AurB (FL-AurB), as indicated. Beta (β)-actin was included as a loading control. Total cell lysate was collected and the levels of Ras, MEK1/2, ERK1/2, hTERT, AurB and 16E6 were ascertained via western blotting. Note the reduced expression of Ras, MEK1/2 and ERK1/2 upon co-expression with either AurB and/or 16E6. **B(ii).** The bar graph shows the mean levels of Ras, MEK1/2 and ERK1/2 quantitated using ImageJ software, and normalized with loading control

in respective experiments. Error bars represent mean ± standard error of mean (SEM) (n=3). (ns = not significant, *P<0.05, **P<0.01, ***P<0.001). **C.** U2-OS cells were transfected with HA-tagged hTERT alone, or with HA-tagged 16E and/or Flag-tagged AurB. The level of telomerase activity upon treatment with 24-hr treatment with 300 nM of AZD1152 was measured using a PCR-based TRAPaze telomerase detection assay. The cell lysate was extracted. Cell extract of HeLa cells or HeLa cells transfected with HA-tagged hTERT were included as a positive control.

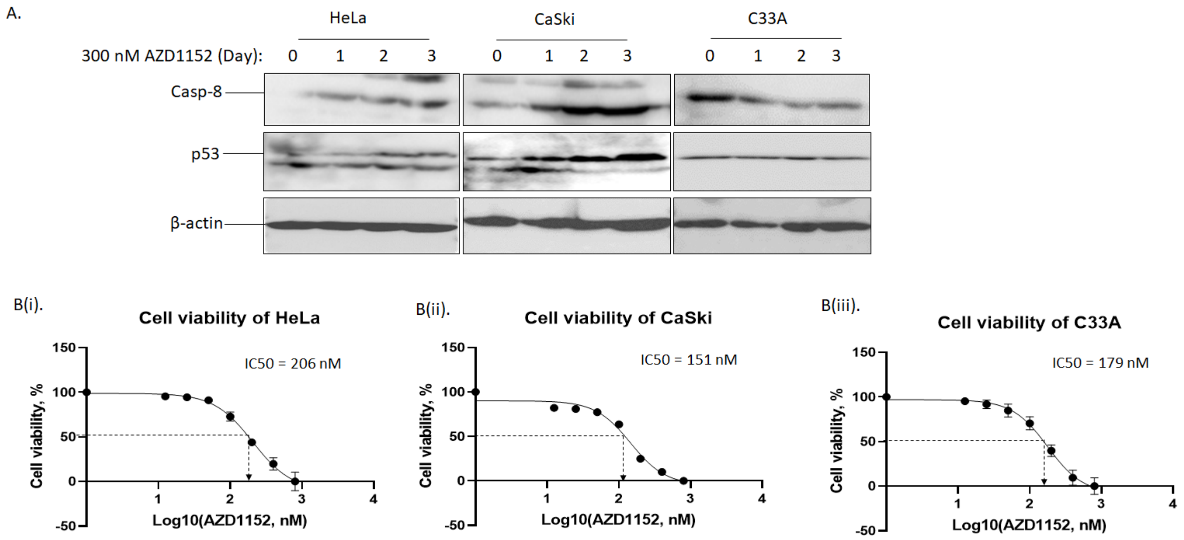

Figure S4. Treatment with AZD1152 led to activation of apoptotic pathway and reduction of cell proliferation. A. The immunoblots show the protein levels of caspase-8 (Casp-8) and p53 in HPV-positve (HeLa and CaSki) and HPV-null (C33A) cancer cells upon 3-day treatment with 300 nM of AZD1152. Beta-actin (β-actin) was included as a loading control. **B.** HPV-positive **(i)** HeLa and **(ii)** CaSki, and -negative **(iii)** C33A cervical cancer cells were treated with 0, 12.5, 25, 50, 100, 200, 400 and 800 nM of AZD1152 for 24 hrs. Cell-counting kit-8 (CCK-8) assay was performed to determine the ability of viable cells to proliferate upon treatment with Aurora kinase inhibitors. Note the CCK8 assays showed the concentration of AZD1152 that reduced the viability of HeLa, CaSki and C33A by half (IC_{50}). The IC_{50} values and graphs were generated using GraphPad Prism. Also shown, is the ability of AZD1152 in inhibiting 50% of the viability of HeLa, CaSki and C33A cells.

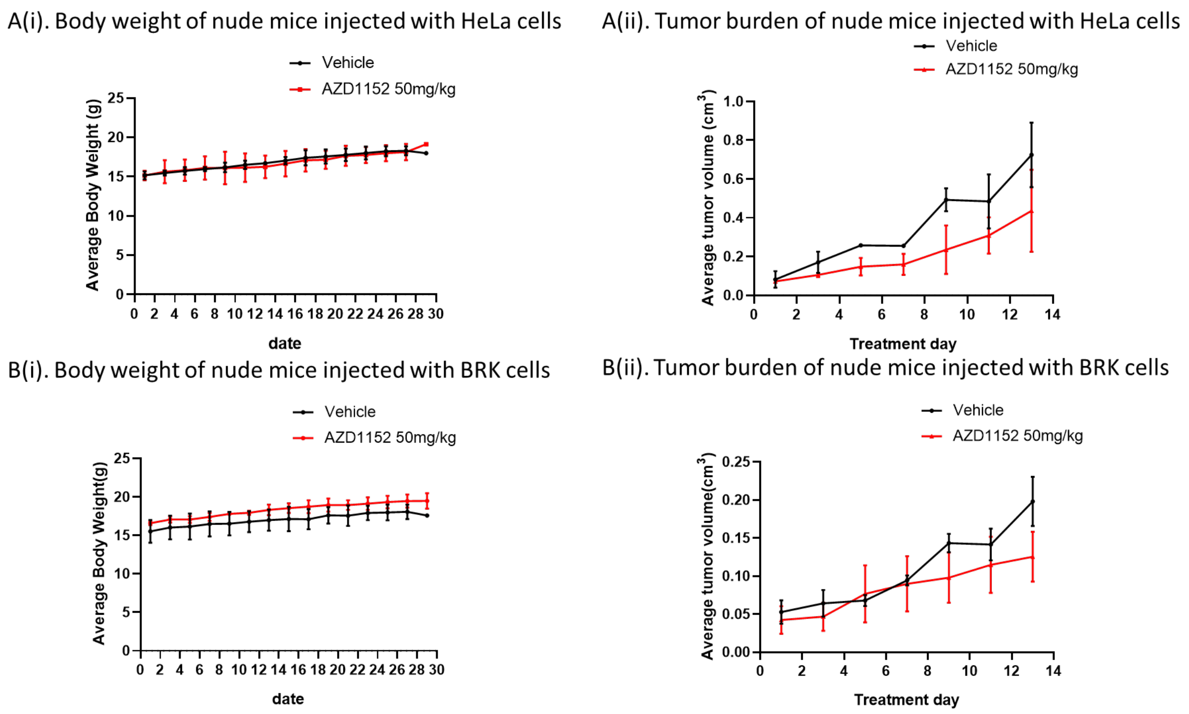

Figure S5. The ability of AZD1152 treatment in inhibiting tumour burden in HPV-bearing and HPV-null athymic nude mice. Relative changes in body weight of athymic nude mice injected with **A(i)** HeLa cells, or **B(i)** immortalized baby rat kidney (BRK) cells were monitored throughout the study period. Tumour growth of the **A(ii)** HeLa or **B(ii)** BRK cells in the mice were monitored every other day throughout the study period. Tumour volume was calculated using the formula $(ab^2) \times 0.5236$.

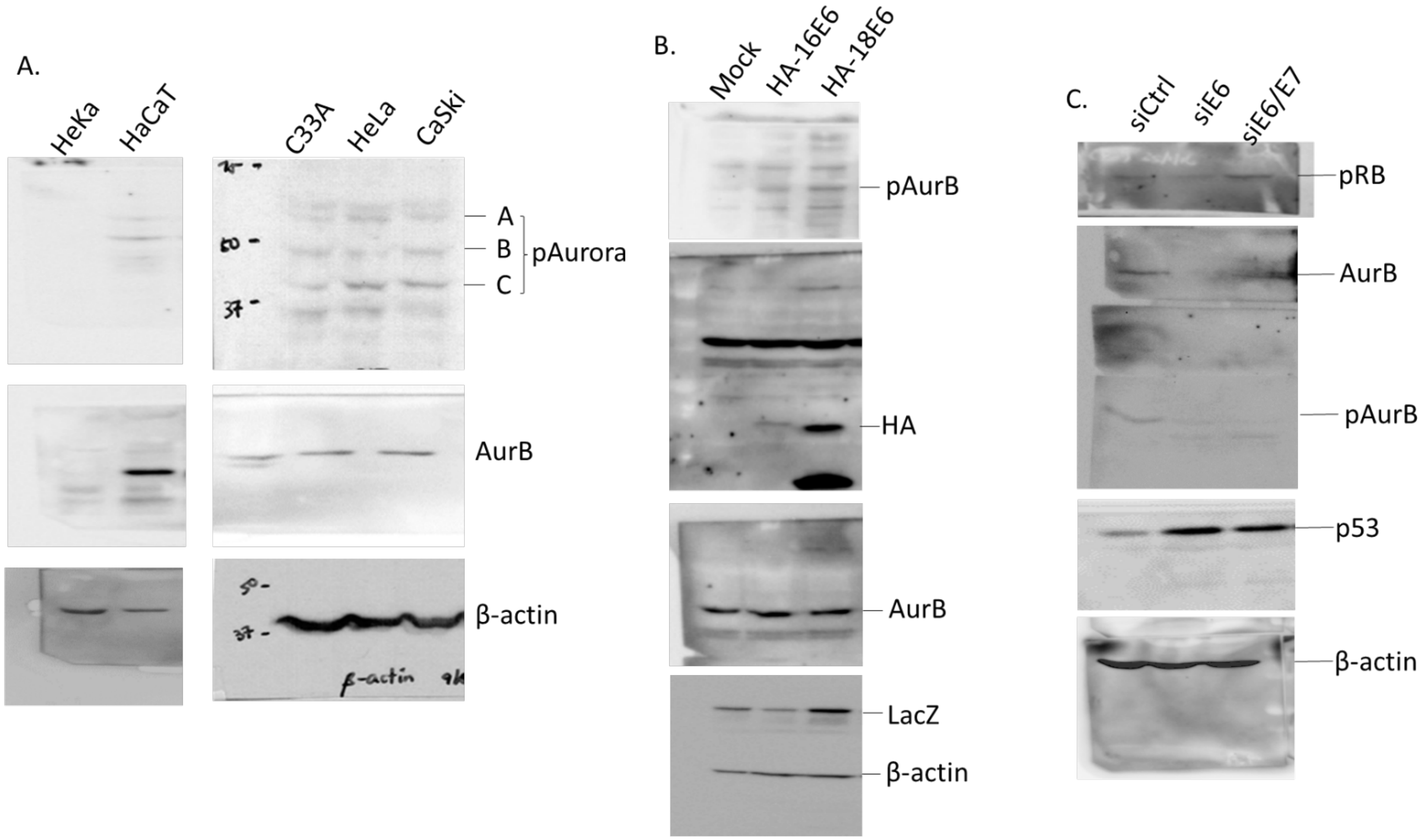

Figure S6. Original immunoblots for Figure 1 showing the level of Aurora kinase B correlated positively with the levels of HPV oncoproteins.

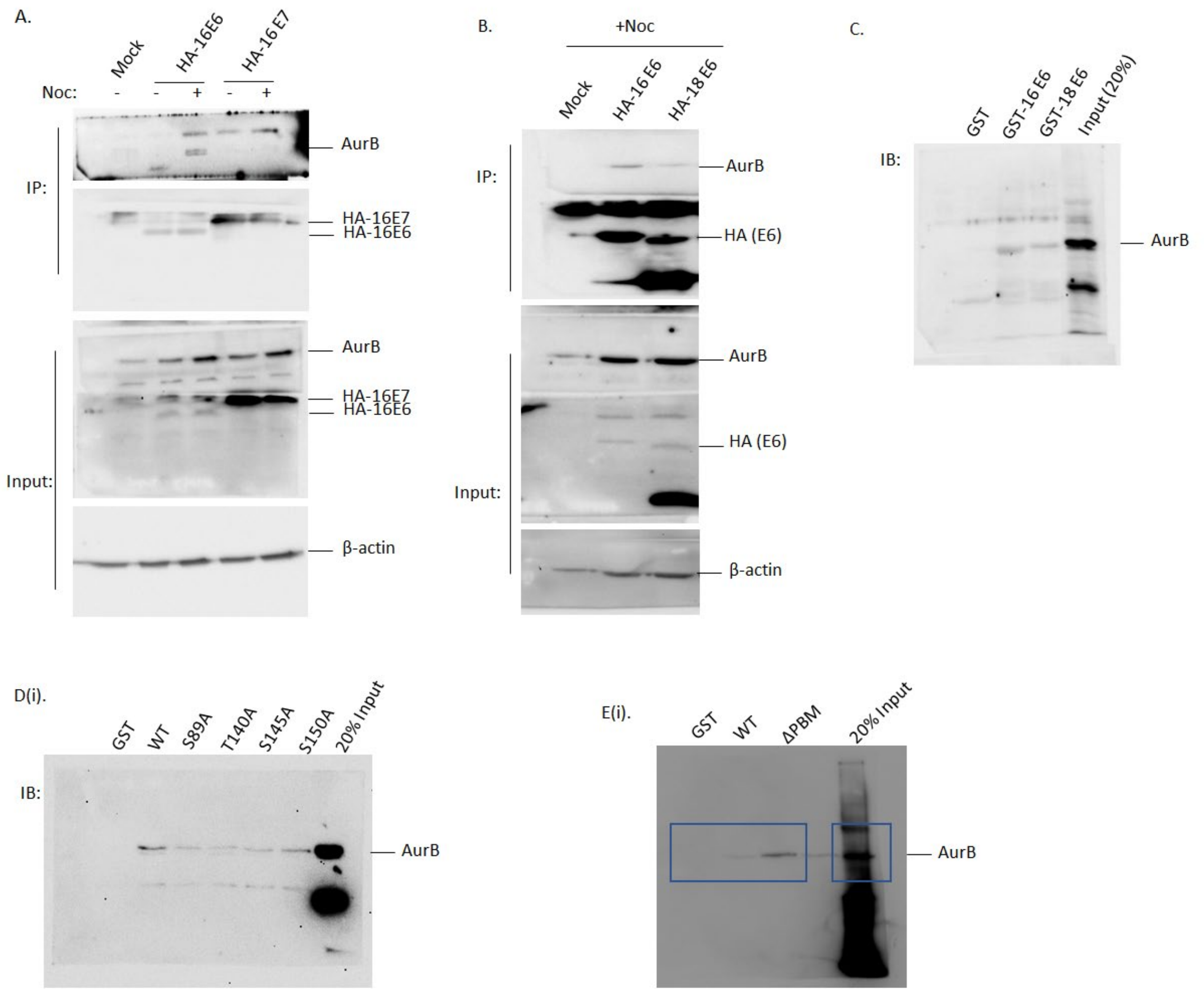

Figure S7. Original immunoblots for Figure 2, showing Aurora kinase B bound directly to the C-terminus of E6-encoded by HPV16 and 18 interacted directly in the nucleus or mitotic cells, leading to perturbation of kinase activity of Aurora kinase B.

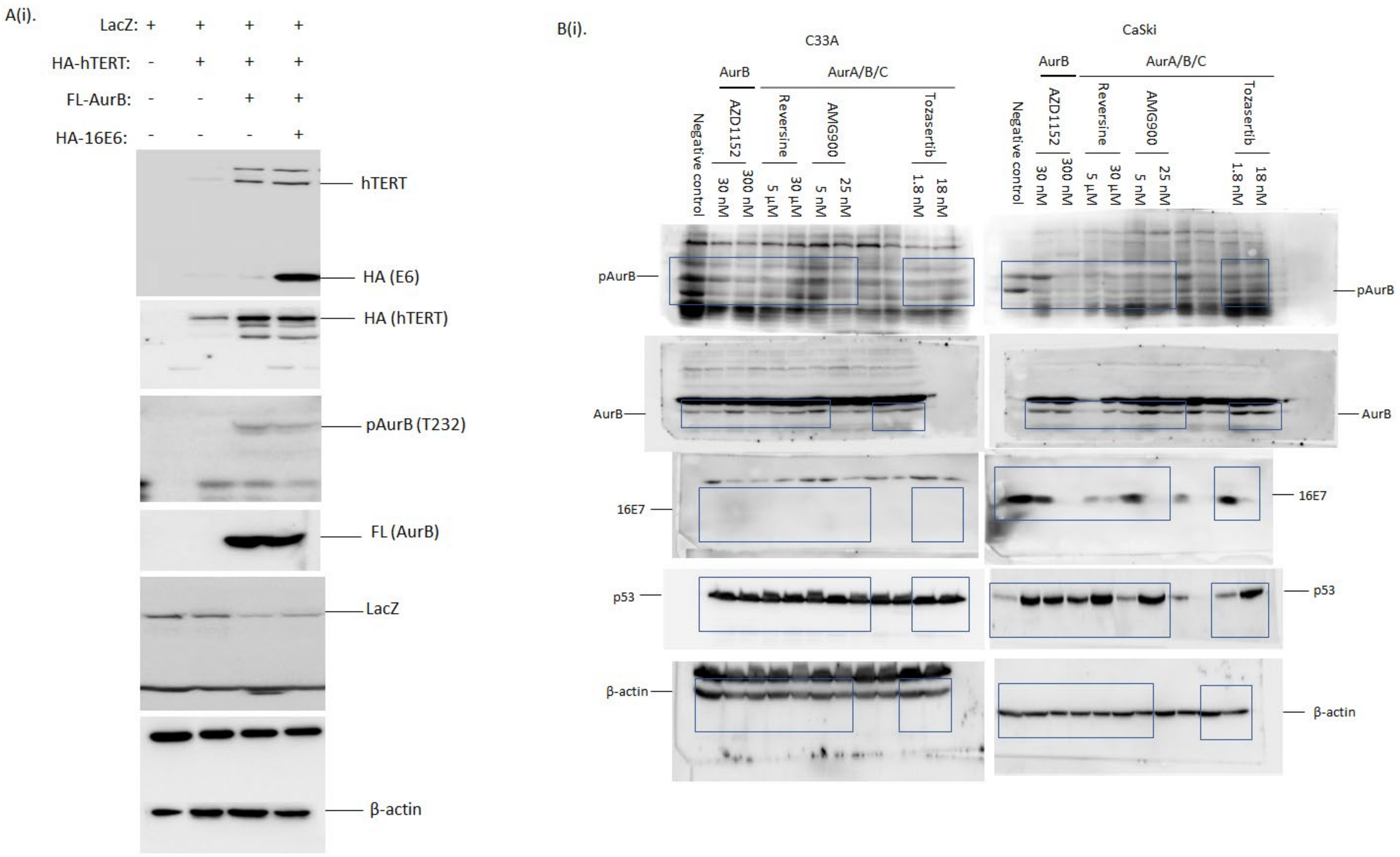

Figure S8. Original immunoblots for Figure 3, showing the levels of human telomerase reverse transcription (hTERT) protein upon expression of Aurora kinase B (AurB) and E6-encoded by HPV16, and the effect of treatment with Aurora kinase inhibitors on the levels of phosphor-AurB, AurB, HPV16E7 and p53.

Table S1: The primer pairs used to generate expression plasmid of HPVE6s that carry intended mutation using the GeneArt^{TM} site-directed mutagenesis. The intended mutation sites are underlined.

PRIMERS	PRIMER SEQUENCES
**16E6 S89A (FWD)**	5’GAGTATAGACATTATTGTTATGCATTGTATGGAACAAC 3’
**16E6 S89A (RVS)**	5’ ATGTTGTTCCATACAATGCATAACAATAATGTCTATAC 3’
**16E6 T140A (FWD)**	5’ AATATAAGGGGTCGGTGGGCAGGTCGATGTATGTCT 3’
**16E6 T140A (RVS)**	5’ CAAGACATACATCGACCTGCCCACCGACCCCTTATA 3’
**16E6 S145A (FWD)**	5’ GGACCGGTCGATGTATGGCATGTTGCAGATCATCAAG 3’
**16E6 S145A (RVS)**	5’ CTTGATGATCTGCAACATGCCATACATCGACCGGTCC 3’
**16E6 S150A (FWD)**	5’ GTCTTGTTGCAGATCAGCAAGAACACGTAGAGAAAC 3’
**16E6 S150A (RVS)**	5’ GTTTCTCTACGTGTTCTTGCTGATCTGCAACAAGAC 3’
